# CTHRSSVVC Peptide as a Possible Early Molecular Imaging Target for Atherosclerosis

**DOI:** 10.3390/ijms17091383

**Published:** 2016-08-24

**Authors:** Rosemeire A. Silva, Ricardo J. Giordano, Paulo S. Gutierrez, Viviane Z. Rocha, Martina Rudnicki, Patrick Kee, Dulcinéia S. P. Abdalla, Pedro Puech-Leão, Bruno Caramelli, Wadih Arap, Renata Pasqualini, José C. Meneghetti, Fabio L. N. Marques, Menka Khoobchandani, Kattesh V. Katti, Ademar B. Lugão, Jorge Kalil

**Affiliations:** 1 Laboratory of Immunology, Heart Institute (InCor), Hospital das Clínicas da Faculdade de Medicina da Universidade de São Paulo, São Paulo 05403-000, Brazil; 2 Chemistry Institute, Biochemistry Department, University of Sao Paulo, Sao Paulo 05508-000, Brazil; giordano@iq.usp.br; 3 Laboratory of Pathology, Heart Institute (InCor), Hospital das Clínicas da Faculdade de Medicina da Universidade de São Paulo, São Paulo 05403-000, Brazil; paulo.gutierrez@incor.usp.br; 4 Clinical Division, Heart Institute (InCor), Hospital das Clínicas da Faculdade de Medicina da Universidade de São Paulo, São Paulo 05403-000, Brazil; vzrocha@hotmail.com (V.Z.R.); bcaramel@usp.br (B.C.); 5 Department of Clinical and Toxicological Analyses, Faculty of Pharmaceutical Sciences University of São Paulo, São Paulo 05508-000, Brazil; martina.rudnicki@gmail.com (M.R.); dspabdalla@gmail.com (D.S.P.A.); 6 Division of Cardiology, Department of Internal Medicine, The University of Texas Health Science Center at Houston, Houston, TX 77054, USA; patrick.kee@uth.tmc.edu; 7 Division of Vascular and Endovascular Surgery, University of São Paulo Medical School, São Paulo 05403-000, Brazil; pedro@puech.com.br; 8 University of New Mexico Comprehensive Cancer Center, Division of Hematology/Oncology and Division of Molecular Medicine, Department of Internal Medicine, University of New Mexico School of Medicine Albuquerque, NM 87131, USA; warap@salud.unm.edu (W.A.); rpasqual@salud.unm.edu (R.P.); 9 Medicine Nuclear Service and Molecular Image, Heart Institute (InCor), Hospital das Clínicas da Faculdade de Medicina da Universidade de São Paulo, São Paulo 05403-000, Brazil; meneghetti@incor.usp.br; 10 Departamento de Radiologia e Oncologia, Faculdade de Medicina da Universidade de São Paulo (LIM43), São Paulo 05403-911, Brazil; fabio.marques@fm.usp.br; 11 Institute of Green Nanotechnology, Department of Radiology and Chemistry, University of Missouri, Columbia, MO 65211, USA; khoobchandanim@health.missouri.edu (M.K.); kattik@health.missouri.edu (K.V.K.); 12 Nuclear and Energy Research Institute—IPEN/CNEN/São Paulo 05508-000, Brazil; ablugao@gmail.com

**Keywords:** atherosclerosis, CD163, macrophages, theranostics, peptide

## Abstract

The purpose of our work was to select phages displaying peptides capable of binding to vascular markers present in human atheroma, and validate their capacity to target the vascular markers in vitro and in low-density lipoprotein receptor knockout (LDLr^−/−^) mouse model of atherosclerosis. By peptide fingerprinting on human atherosclerotic tissues, we selected and isolated four different peptides sequences, which bind to atherosclerotic lesions and share significant similarity to known human proteins with prominent roles in atherosclerosis. The CTHRSSVVC-phage peptide displayed the strongest reactivity with human carotid atherosclerotic lesions (*p* < 0.05), when compared to tissues from normal carotid arteries. This peptide sequence shares similarity to a sequence present in the fifth scavenger receptor cysteine-rich (SRCR) domain of CD163, which appeared to bind to CD163, and subsequently, was internalized by macrophages. Moreover, the CTHRSSVVC-phage targets atherosclerotic lesions of a low-density lipoprotein receptor knockout (LDLr^−/−^) mouse model of atherosclerosis in vivo to High-Fat diet group versus Control group. Tetraazacyclododecane-1,4,7,10-tetraacetic acid-CTHRSSVVC peptide (DOTA-CTHRSSVVC) was synthesized and labeled with ^111^InCl_3_ in >95% yield as determined by high performance liquid chromatography (HPLC), to validate the binding of the peptide in atherosclerotic plaque specimens. The results supported our hypothesis that CTHRSSVVC peptide has a remarkable sequence for the development of theranostics approaches in the treatment of atherosclerosis and other diseases.

## 1. Introduction

Atherosclerosis is a chronic inflammatory disorder in which inflammatory cells accumulate and interact with various components within the arterial wall [1]. Although the initial atherosclerotic lesions are asymptomatic, the progression of the disease results in the development of unstable plaques that are prone to rupture, and results in arterial occlusion and ischemic damage [2]. Macrophages are important inflammatory cells implicated in the pathogenesis of atherosclerotic lesions. Specifically, macrophage subtypes play several roles in the progression and destabilization of the plaques [3]. Stimuli such as cytokines, modified lipids, proteolytic enzymes and heme appear to contribute to the progression of vulnerable lesions [1,4]. Furthermore, the atheroma microenvironment can also modify the functional phenotypes of macrophages [3]. Intraplaque hemorrhage might activate the differentiation of monocytes to a macrophage subtype known as hemoglobin (Hb)-stimulated macrophage (M(Hb)) [4]. The surface of these M(Hb) displays receptors such as the scavenger receptor cysteine-rich type 1 protein M130 (CD163) and macrophage mannose receptor 1 (MMR or CD206) [3]. From a pathological perspective, CD163 receptor binds to the hemoglobin:haptoglobin (Hb:Hp) complex, and mediates its endocytosis and subsequent clearance [5,6]. Accordingly, this interplay between cellular mechanisms and molecular factors affects the development and the pathophysiological consequences of atherosclerosis. Although different cellular mechanisms and molecular factors that trigger the development of atherosclerosis have been identified [2], the chronic nature of the atherothrombotic vascular disease and its unpredictability are frequently a life-threatening condition for which prognostic and therapeutic options are limited. The main objective of our work was to select phages displaying peptides capable of binding to vascular markers present in human atheroma as well as validate its targeting properties using in vitro assays and a low-density lipoprotein receptor knockout (LDLr^−/−^) mouse model of atherosclerosis.

Accurate molecular imaging and therapeutic probes require site specific and selective binding with the receptors expressed by target cells. The past decade has seen considerable progress on the application of phage display technology for the development of receptor-specific peptides and a variety of epitopes of high specificity in ligand binding for diagnosis and therapy of various diseases including atherosclerosis [7,8,9,10,11]. A number of recent studies have demonstrated that phage display is an ideal platform for the identification of vascular targets for probing the vasculature beyond the receptor expression levels [12,13]. This approach is highly attractive in molecular imaging and therapy because it allows direct accessibility into the molecular targets ligand-directed pharmacodelivery.

## 2. Results

### 2.1. Selection of Phages Displaying Peptides Bound to Atherosclerotic Lesions

**Figure 1:**
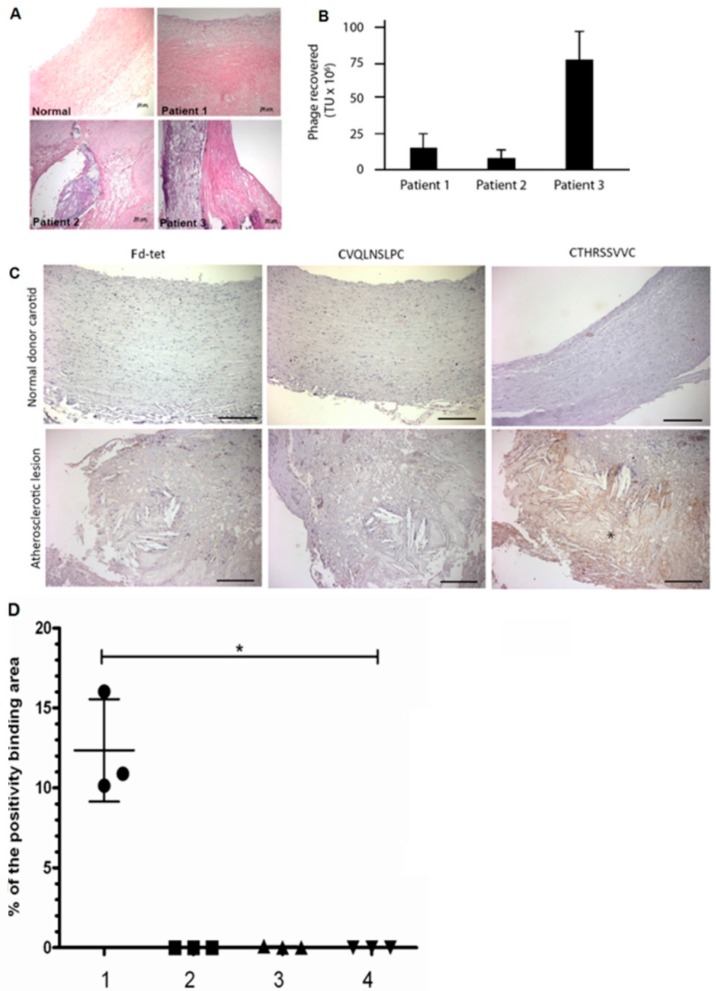
Phages displaying the peptide sequence CTHRSSVVC bind to human atherosclerotic lesions. (**A**) hematoxylin–eosin (HE) staining to human samples. Bar, 50 µm in all panels; (**B**) Phage recovery from CX7C peptide phage library after each round of biopanning in atheroma from each patient. Error bars indicate standard error of mean (SEM); (**C**) Phage Overlay Assay to atheroma and normal donor carotid tissue samples (*n* = 3, each group), figure shows representative slides of immunohistochemical staining for CTHRSSVVC-phage (CD163-mimic) and CVQLNSLPC-phage (Galectin-4 mimic) in the normal carotid (**top**) and atherosclerotic lesion from patient 2 (**bottom**), with a strong positively stained to lipid core region (hemorrhage, thrombus and cholesterol crystals) are indicated by the star (*). Bar, 100 µm in all panels; (**D**) Quantification of binding phage in human atheroma and normal carotid (*n* = 3, each group), was carried out in twelve samples that were divided in four different groups: (**1**) Atheroma with CTHRSSVVC-phage; (**2**) Atheroma with Fd-phage; (**3**) Normal carotid with CTHRSSVVC-phage; and (**4**) Normal carotid with Fd-phage. Statistical difference was determined by one-way analysis of variance followed by the Kruskal–Wallis test, and it was accepted at a value of * *p* < 0.05, vs. normal carotid.

We have used a phage display library (labeled as CX7C) in which seven amino acid long random peptides are displayed on the surface of a filamentous phage fused to the minor pIII coat protein [8]. Given the heterogeneity of atherosclerosis disease, and our goal to develop target-specific peptides that bind to molecular markers which are common to most atherosclerotic lesions, phage display selection was performed in tandem using specimens obtained from three different patients whose atheroma had distinct inflammation characteristics to the lipid core region, (Figure 1A, Table 1). Although the initial histologic analyses were not complete, all elements of the intima, including those from the atherosclerotic lesion and endothelium, were exposed to the procedures.

Collectively, the three samples displayed key features of atherosclerosis: inflammation, thrombus, calcium deposits, and hemorrhage. Phages bound to the luminal surface of the lesion of the patient 1 were, therefore, amplified overnight and incubated with the sample from patient 2, and so on. After three rounds of selection, we observed a 5.6-fold enrichment of phage binding to the specimen from patient 3 compared to the number of phages bound to the sample obtained from patient 1 (Figure 1B, Table 1). We infer this observation to the diversity of atheroma components in the specimens.

### 2.2. Peptide Selection and Receptor Identification

After three rounds of selection, individual phage clones were randomly picked for sequencing and identification of the peptides displayed on the surface of the bacteriophage particles. The sequencing results indicated that all 49 phage clones displayed distinct peptides. This was not unexpected, considering the complexity of our sample and the number of possible molecular targets expressed in atherosclerotic lesions. This observation was also in good agreement with previous screening studies using phage display on cells [14] and also in in vivo models [8]. To prioritize peptides among our 49 individual phage clones identified by sequencing, we performed further analysis using a combination of bioinformatics and a functional assay (phage overlay). Both approaches have been successfully utilized to isolate and validate peptides relevant in other biological contexts such as angiogenesis, pulmonary diseases, and cancer [8,14,15]. First, we performed a database search to identify peptides sharing significant homology with known proteins, in particular, human proteins involved in atherosclerosis or other diseases with a vascular or immunological component. Among the putative receptors identified by bioinformatics, placental growth factor (PlGF) and the macrophage expressed protein (CD163), two possible prognostic markers for disease progression and plaque rupture, were identified [16,17]. Thus, we have selected phage displaying the peptides CVSSTLLRC (PlGF-mimic) and CTHRSSVVC (CD163-mimic) for further validation. Two additional peptides CVQLNSLPC (Galectin-4 mimic) and CQAYKLGSC (Collagen α-1 (IX) chain-mimic) were also selected (Table 2). Subsequently, phage overlay assays were performed using the phage particles as a surrogate of the peptides.

### 2.3. CTHRSSVVC-Phage Targets for Tissues

**Figure 2:**
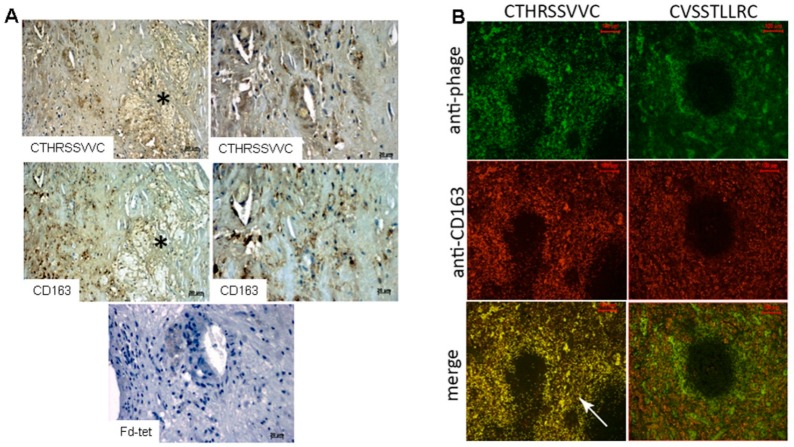
CD163 expression and CTHRSSVVC-phage overlay by different assays co-localize in atheroma and spleen tissues. (**A**) CTHRSSVVC-phage overlay localization and expression of CD163 in atherosclerotic tissue particularly in areas rich in macrophages (indicated by the star (*)). Phages bound to tissue were revealed using an anti-bacteriophage antibody. No significant staining was observed with the insertless phage Fd-tet. Bar, 50 µm (**left** panels) and 20 µm (**right** panels); (**B**) CTHRSSVVC (CD163-mimic) or CVSSTLLRC (PlGF-mimic) phage localization (**green**) and CD163 expression (**red**) in spleen tissue observed by immunofluorescence and visualized by confocal microscopy. Phages bound to tissue were revealed using an anti-bacteriophage antibody. The bottom panels show the merge of the phage overlay (**upper** panels) and CD163 expression (**middle** panels). Bright yellow (as exemplified by the arrows) indicates co-localization. Note that the co-localization with CVSSTLLRC-phage was not as complete as with the other peptide. Bar, 100 µm in all panels.

**Figure 3:**
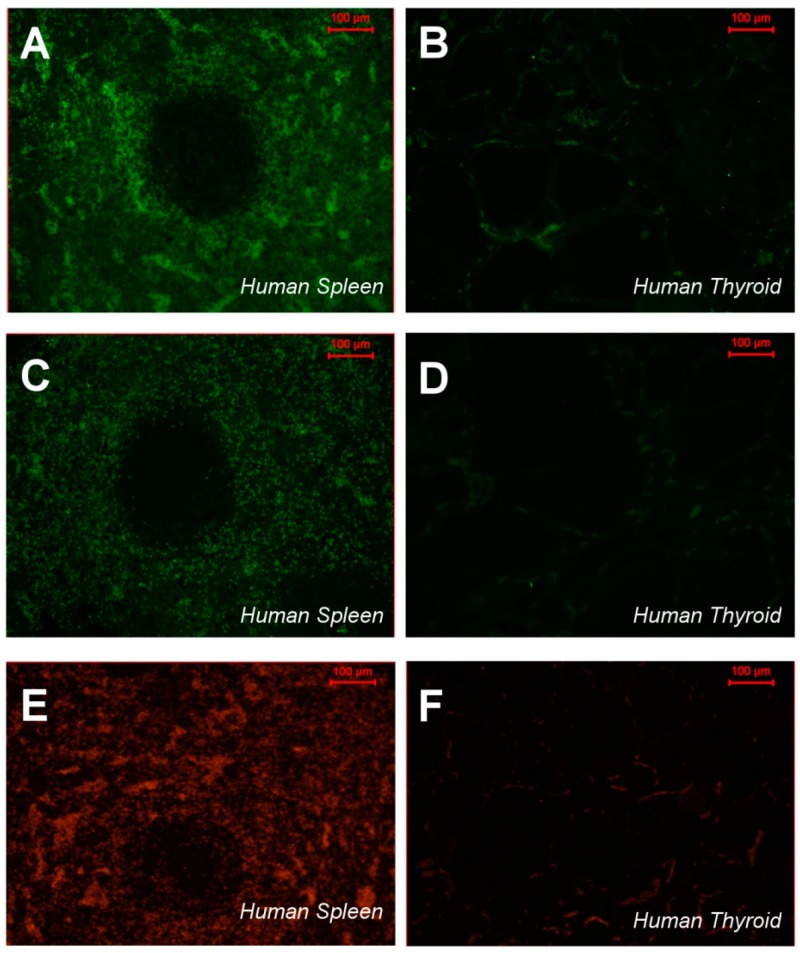
Immunofluorescence to CTHRSSVVC-phage, Fd-phage and CD163 expressions: (**A**,**B**) CTHRSSVVC-phage localization (**green**) to spleen and thyroid; (**C**,**D**) Fd-insertless phage localization (**green**) to spleen and thyroid; and (**E**,**F**) CD163 expression (**red**) to spleen and thyroid respectively, revealed by immunofluorescence and photographed with an AxioVision (Carl Zeiss Inc., Jena, Germany) microscope. Phages bound to tissue were revealed using an anti-bacteriophage antibody. The thyroid tissue (negative control), the reactions resulted negative. Bar, 100 µm in all panels.

The phage overlay studies showed that three peptides had low reactivity in atherosclerotic lesions (Table 1), whereas the CTHRSSVVC-phage peptide presented strong positivity in the same sample and lipid core region from three patients (Figure 1C, Table 1), although a simple background for phages could also be observed (Figure 1C). As a proof of specificity, control experiments demonstrated no reactivity between CTHRSSVVC-phage peptide and normal carotid samples, and between negative control insertless phage (Fd-tet) and atherosclerotic carotid samples. The analysis of the results from bioinformatic and functional assays (phage overlay) showed that CTHRSSVVC-phage peptide mimic proteins with relevant roles in atherosclerosis progression and had the strongest reactivity against atherosclerotic lesions. The CTHRSSVVC-phage peptide was selected for further studies (Figure 1C). Quantification of binding phage in human atheroma and normal carotid was carried out in twelve samples that were divided in four different groups: (1) Atheroma with CTHRSSVVC-phage; (2) Atheroma with Fd-phage; (3) Normal carotid with CTHRSSVVC-phage; and (4) Normal carotid with Fd-phage (Figure 1D, Table 3). CD163 expression and CTHRSSVVC-phage overlay were co-localized in atheroma and spleen tissues, respectively. The significant overlap in tissue staining by CTHRSSVVC-phage (Figure 2A) and anti-CD163 (Figure 2A) were observed in atherosclerotic lesions, particularly, in areas rich in macrophages (Figure 2A, indicated by the star (*)). No significant staining was detected when the Fd-tet insertless phage (Figure 2A) or control antibody were utilized. However, single background populations for phages were observed. We have further modified the phage overlay protocol, and it was done by immunofluorescence and fluorescence microscopy to allow for better overlap of the images. Consequently, the merger of the two channels would indicate a perfect co-localization of phage and CD163 staining. Indeed, as shown in Figure 2B, CTHRSSVVC-phage overlay (green) and CD163 immunostaining (red) overlap produced an almost entirely yellow image. The co-localization observed when the phage displaying a different peptide (CVSSTLLRC) was incubated with the spleen tissue (Figure 2B) was less complete. When the tissues were immunostained with the thyroid tissue (negative control), the reactions resulted in negative observation (Figure 3).

### 2.4. Identification and Characterization of a CD163 Binding Peptide

**Figure 4:**
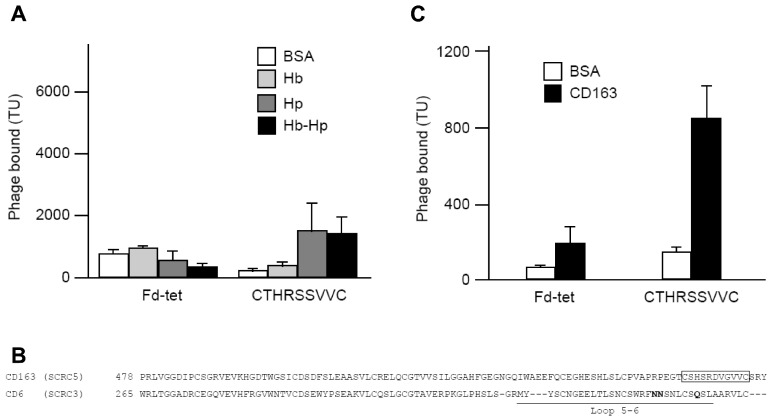
Phage binding to immobilized ligands. (**A**) Binding of CTHRSSVVC or control Fd-tet phages to hemoglobin (Hb), haptoglobin (Hp), the HbHp complex or bovine serum albumin (BSA); (**B**) Alignment of fifth and third SRCR domains of CD163 and CD6, respectively. The box indicates the region in CD163 that shares similarity to CTHRSSVVC; underlined is the fifth and sixth loop highly divergent region. Amino acids essential for CD6 binding to CD166 are also indicated (**bold**); (**C**) Binding of CTHRSSVVC or control Fd-tet phages to immobilized CD163 or BSA. The data are averages of triplicates and are expressed as mean ± SEM.

**Figure 5:**
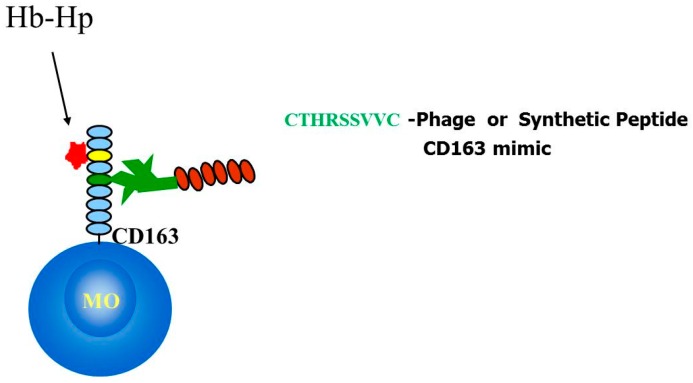
YES Hypothesis in situ. Fifth Scavenger Receptor domain of CD163 probably is pseudo-homodimer with CTHRSSVVC peptide (CD163-mimic).

The selected peptide CTHRSSVVC shares 78% homology and 67% identity with human CD163 (Table 2). CD163, also known as scavenger receptor cysteine-rich type 1 protein or M130, is preferentially expressed by monocytes and macrophages, and the best characterized function is the clearance of hemoglobin in the form of haptoglobin-hemoglobin complex [17,18,19]. The role of CTHRSSVVC peptide in atherosclerosis is possibly related with macrophage activation and the production of anti-inflammatory cytokines [19]. It is also a potential serum marker for atherosclerosis disease progression [16,20]. However, the peptides identified by phage display are often biologically active and interact with relevant binding sites [7,8,12,14]. Therefore, we decided to evaluate whether CTHRSSVVC-phage could bind to the native ligand of CD163. In this study, hemoglobin (Hb), haptoglobin (Hp) and hemoglobin-haptoglobin complex (HbHp) were immobilized on microtiter plates, and incubated with CTHRSSVVC or Fd-tet insertless negative control phages. As observed in Figure 4A, no significant binding of CTHRSSVVC-phage to BSA or Hb was detected. We therefore hypothesized that CTHRSSVVC might mimic a domain that participates in receptor homodimerization; indeed previous studies have suggested that CD163 dimerization is important for signaling and activation [21] (Figure 5). To validate this hypothesis, CD163 was immobilized on microtiter plates and incubated with CTHRSSVVC or Fd-tet negative control phages. Our experimental results validated our hypothesis as they unequivocally confirmed that the phage displaying the CTHRSSVVC peptide indeed binds to immobilized CD163 as depicted in Figure 4C. It was also interesting to observe that CTHRSSVVC shares similarity to the “fifth and sixth loop” regions of the SRCR domain, a highly divergent region proposed to be involved in ligand binding [22] (Figure 4B). These data corroborate with the results, which showed that the Hb-Hp binding site is located in the third scavenger receptor cysteine-rich (SRCR) domain of CD163 [5,6], while CTHRSSVVC shares similarity to a sequence present in the fifth SRCR domain of CD163 (Figure 4B). Moreover, site-directed mutagenesis showed that the amino acid residues within the fifth and sixth loop region of the SRCR domain of another scavenger receptor (CD6) are essential for binding to its ligand (CD166) [23].

In summary, our data suggest that the atheroma binding peptide sequence CTHRSSVVC is a versatile CD163-mimic peptide that maps to a putative homodimerization domain in this multidomain scavenger receptor molecule. Our results also provide experimental evidence of a potential role for the fifth SRCR domain of CD163 and CTHRSSVVC peptide sequence (Figure 4B).

### 2.5. CTHRSSVVC-Phage Is Internalized by CD163 Expressing Cells

**Figure 6:**
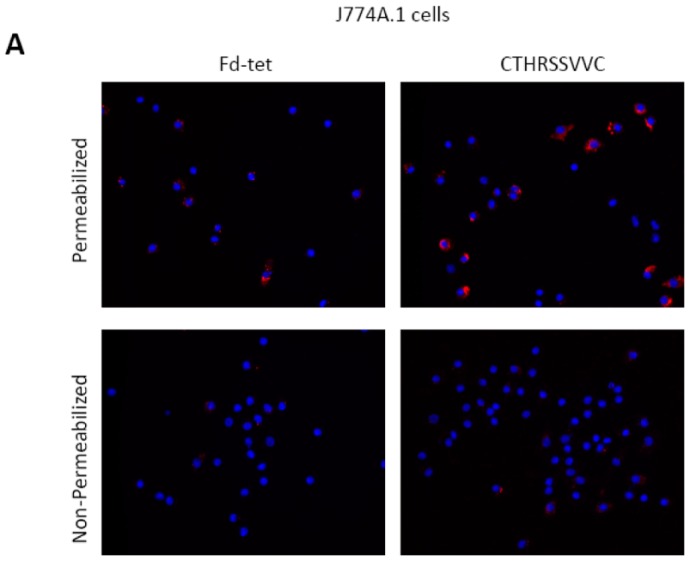

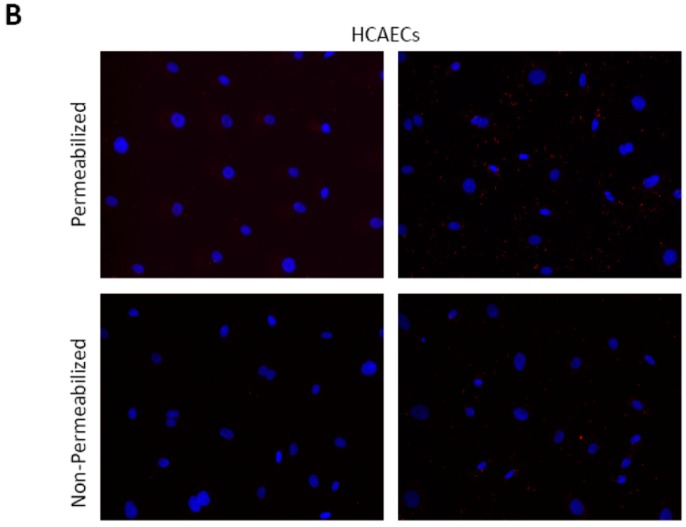
CTHRSSVVC-phage is internalized by macrophages. CTHRSSVVC-phage is internalized by J774A.1 mouse macrophage but not human endothelial cells (HCAECs). J774A.1 cells (**A**) or HCAECs (**B**) were incubated at 37 °C with CTHRSSVVC or insertless Fd-tet phages for 8 h to allow phage internalization. Membrane bound phages were removed by cell washing, internalized phages (red) and cell nucleus (blue) revealed by cell permeabilization, and phage staining using an anti-bacteriophage antibody. Non-permeabilized cells indicate the successful removal of cell surface bound phages. Magnification = 100×.

Encouraged by our results that the CTHRSSVVC-phage targets CD163 positive cells, we then probed whether this peptide could participate in receptor-mediated internalization. Selected peptides that target membrane bound receptors can be internalized by cells in a receptor-dependent manner [15,24]. We have therefore evaluated the capability of phage displaying the CTHRSSVVC peptide for internalization by macrophages. In these experiments, we selected a CD163^+^ mouse derived macrophage like monocyte cell line (J774A.1 cells) to perform our assay [25]. As a control, we used human coronary artery endothelial cells (HCAECs) because they do not express CD163 receptors. Cells were seeded on microtiter wells and incubated with CTHRSSVVC or Fd-tet insertless negative control phages for 8 h. The cells were then washed to remove all surface bound phage and internalized phage particles visualized by immunofluorescence using permeabilized cells. We found that CTHRSSVVC-phage was efficiently internalized by the J774A.1 cells (Figure 6A) but not by HCAECs (Figure 6B). Because macrophages are professional phagocytic cells, we observed that both phages (CTHRSSVVC and insertless Fd-tet) were internalized to a certain extent by the J774A.1 cells in sharp contrast to the human coronary endothelial cells. However, it is important to note that the CTHRSSVVC-phage was internalized by J774A.1 cells to a significantly larger extent as compared to the negative insertless control phage. In all cases, non-permeabilized cells were negative, indicating that no surface bound phage remained after the washing steps. These experimental results, taken together, suggest that CTHRSSVVC synthetic peptide is also internalized by CD163^+^ macrophages through receptor-mediated endocytosis (Unpublished data).

### 2.6. CTHRSSVVC-Phage Targets Atherosclerotic Lesions in Vivo

Having characterized ligand-directed atheroma binding in vitro, we then considered assessing the ability of the CTHRSSVVC-phage to target atherosclerotic lesions in vivo. Circulation time is an important variable to consider for in vivo phage targeting experiments because it represents the time that phage is allowed to circulate after intravenous administration, thereby influencing biodistribution. Therefore, experiments can be designed to detect phage binding to the vascular endothelium (5–10 min of circulation before phage recovery) or to detect phage that had been internalized by the endothelium or other non-endothelial tissue layers (circulation for 24 h before phage recovery) [26,27]. In this study, post 24 h of circulation, the CTHRSSVVC-phage was enriched and confirmed by immunohistochemical staining of phage in tissue sections.

**Figure 7:**
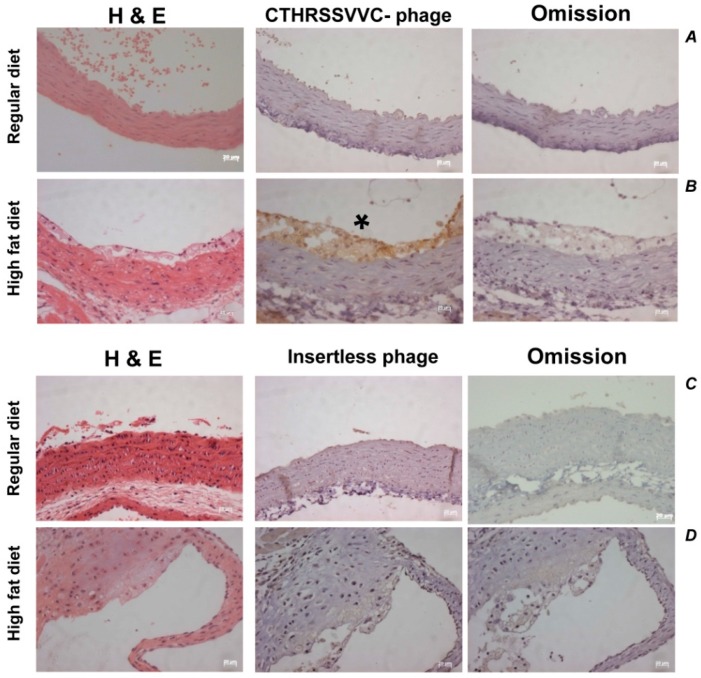
CTHRSSVVC-phage to Regular (**A**) and High-Fat diet (**B**); and insertless phage to regular (**C**) and High-Fat diet (**D**); homing specificity to organ target. Representative sections of mice aortic root from Regular (*n* = 8) and High-Fat diet (*n* = 13) groups. Hematoxylin–eosin (HE) staining to aorta was performed to all groups. High-Fat diet group presented a strong positively stained area as indicated by the star (*) to CTHRSSVVC-phage homing (**B**) and negative to insertless Fd-tet phage on the same diet (**D**). The CTHRSSVVC-phage bound quantification in atheroma of High-Fat diet mice (**B**) was reported as means ± SEM 45.50, ±S.D 13.97 and all other sections were negative to phage homing. As a reaction control, primary antibody was omitted. Bar, 20 µm in all panels.

A strong positive phage staining area was observed in the atherosclerotic tissue sections of all LDLr^−^/^−^ mice fed with a High-Fat diet that received the CTHRSSVVC-phage injection (Figure 7, marked by the star (*)). However, no positivity was detected in the aorta from the LDLr^−^/^−^ mice fed with a Regular-diet. Although background for phages was observed in aorta and atherosclerotic lesions from LDLr^−^/^−^ mice fed on both Regular and High-Fat diet that received the insertless phage injection, this staining was not significant. As a reaction control, primary antibody was omitted in adjacent sections, and hematoxylin–eosin (HE) staining was performed in all samples (Figure 7).

Our experimental findings have clearly revealed that the CTHRSSVVC-phage targets atherosclerotic lesions in vivo (Figure 7). The statistical significance of experimental observations was determined by Fisher´s exact test at a value of *p* < 0.01 as statistically significant (Table 4). The CTHRSSVVC-phage bound quantification to atheroma of High-Fat diet mice was ± standard error of mean (SEM) 45.50, ±S.D 13.97, the insertless phage control group in this diet as well as to Regular-diet group with CTHRSSVVC-phage and insertless phage were negative as confirmed by semi-quantitative analysis.

### 2.7. ^111^In-DOTA-CTHRSSVVC Peptide Preparation and Atheroma/Normal Carotid Binding Studies

Labeling of ^111^In-tetraazacyclododecane-1,4,7,10-tetraacetic acid-CTHRSSVVC peptide (^111^In-DOTA-CTHRSSVVC) gave radiochemical yield higher than 95%, as determined by high performance liquid chromatography (HPLC). The binding study, using the same samples that were selected for phage overlay, indicated an uptake in atheroma with 210 counts/mg, whereas in normal carotid the value was 107 counts/mg, representing a ratio of 96% higher in atheroma as compared with carotid. In the blocking experiments, using just atheroma, the solution without the cold peptide gave an uptake equal to 440 counts/mg, whereas in solution with cold peptide the value was 260 counts/mg, representing a decrease of 41% in the blocked experiment.

## 3. Discussion

One of the great challenges in atherosclerosis is the identification of molecular markers with signatures of predicting plaque vulnerability. Identifying patients at high risk of a cardiovascular event might be achieved by serum marker levels or by new and improved imaging protocols capable of distinguishing stable from unstable plaques [26,28]. In this study, we reasoned that further understanding of the molecular profile of atherosclerotic lesions could reveal important clues for improved targeted therapies and novel imaging procedures to evaluate patients at risk of acute vascular events. Therefore, we fingerprinted human atherosclerotic plaques of a phage display peptide library and identified several peptides that bind to distinct areas in the atheroma after three rounds of selection. We have utilized a combination of bioinformatics and functional assay (phage overlay) in our efforts to select peptides with promising applications in the diagnosis and therapy of atherosclerosis. Both these approaches have been successfully utilized in identifying peptides sharing significant homology with known proteins, and to isolate and validate peptides relevant in a myriad of biological contexts such as angiogenesis, pulmonary diseases, and cancer [8,11,14,15,18,19].

Among the peptides selected and identified by bioinformatics were phages displaying the peptides CVSSTLLRC (PlGF-mimic), CTHRSSVVC (CD163-mimic), CVQLNSLPC (Galectin-4 mimic) and CQAYKLGSC (Collagen α-1 (IX) chain-mimic). One of the selected peptides mimics a putative CD163 homodimerization domain and considering that CD163 has a prominent role in inflammation, this CTHRSSVVC-phage showed overall the strongest reactivity and binding affinity to atherosclerotic lesions and has been validated as a potential marker for atherosclerosis disease progression [20,21]. Its role in atherosclerosis is possibly related with macrophage activation and the production of anti-inflammatory cytokines [19]. Therefore, CTHRSSVVC synthetic peptide sequence is an important candidate as a targeting agent for further development with potential application in molecular imaging and therapy of atherosclerosis and various CD163 positive diseases.

Given that the phage display selection was performed in tandem using specimens obtained from three different patients. The atheroma of these patients had distinct inflammation with and without thrombus and hemorrhage characteristics, at the lipid core regions. We have observed statistical significance at quantifying the bound phage positivity, which reinforces the enrichment data from patient 3 compared to the number of phages that bound to the sample obtained from patient 1. These observations could be attributed to the different atheroma cells compositions in the individual patients. It is well-known that atherosclerosis is associated with the M1-type state, and therefore it is beneficial to determine how macrophage polarity influences phage homing and uptake both in vitro and in vivo.

It may be noted that CD163 is a specific macrophage activation marker since it is exclusively expressed on the cell surface of monocytes and macrophages. It is also a potential serum marker for atherosclerosis disease progression besides its role in the clearance of hemoglobin in the form of haptoglobin-hemoglobin complex by the third scavenger receptor cysteine-rich (SRCR) domain of CD163 [20]. Remarkably, the peptide sequence CTHRSSVVC evaluated in our current work, shares similarity to a sequence present in the “fifth and sixth loop” region of the SRCR domain. Notably, the phage displaying the CTHRSSVVC peptide also binds to immobilized CD163. Experimental evidence suggests that CD163 dimerization is important for signaling and activation, [25]. We therefore hypothesized that CTHRSSVVC peptide will mimic a domain that participates in receptor homodimerization, a highly divergent region proposed to be involved in ligand binding [22,25]. Detailed in vivo investigations in animal models will be necessary to validate the potential role of this peptide sequence CTHRSSVVC that shares similarity to a sequence present in the fifth SRCR domain of CD163. Such studies will provide important insights for using CTHRSSVVC peptide as a target specific vector to diagnose atherosclerosis and other CD163-positive diseases. This peptide also presents new opportunities in drug design as it can be used to attract macrophages to the site of partially dissolved thrombus, thus effecting efficient clot dissolution. This approach provides an alternative clearance gateway that might be exploited in anti-inflammatory therapy. Our detailed in vitro and in vivo investigations, as elaborated above, have conclusively demonstrated that CTHRSSVVC-phage peptide is internalized, in excellent propensity, by CD163-expressing cells which are widely present in plaques, thus unraveling novel therapeutic possibilities in cardiology.

Our in vivo investigations using LDLr^−/−^ mice injected with CTHRSSVVC-phage, have demonstrated strong atheroma positivity when compared to the control group thus confirming specific homing to the atherosclerotic lesions. These studies have therefore laid a solid foundation and proof of concept confirming that the CTHRSSVVC synthetic peptide as an important candidate for its use as a target specific agent in molecular imaging and therapy of atherosclerosis.

Successful efforts to label DOTA-CHTRSSVVC peptide with ^111^In, to produce ^111^In-DOTA-CTHRSSVVC peptide agent, will serve as a new molecular imaging tool to probe the atheroma for drug delivery and also in the evaluation of drug efficacy in clinical trials and treatment regimens [29,30]. Although ^111^In is not the best radionuclide for imaging purposes, we have obtained the SPECT images of excised organs and performed autoradiography of normal and hypercholesterolemic diet-fed LDLr^−/−^ mice arteries in this preliminary pilot investigation. The autoradiography experiments clearly demonstrated clinically discernable and useful uptake patterns (Figure S1). New imaging experiments using superior single-photon emission computed tomography (SPECT) radionuclides will be done in the near future.

The results of our investigations have unraveled the role CTHRSSVVC peptide sequence to bind CD163 receptors, and therefore, present new opportunities in drug design and as drug delivery vehicles capable of selective binding to atherosclerotic plaques and to target cell surface receptors in vivo [31,32,33,34,35,36].

## 4. Materials and Methods

### 4.1. General Procedures

#### 4.1.1. Ethics Statement

This study has been approved by the Scientific and Ethical Committee from the Heart Institute (InCor), Hospital das Clínicas da Faculdade de Medicina da Universidade de São Paulo, São Paulo, Brazil (protocol number 054/2003, 19 February2003) and all the selected patients, as well as an organ donor subject or legal next of kin, provided written informed consent. The clinical investigation was performed based on the standards of the Declaration of Helsinki.

The animal procedures to this study were reviewed and approved by the Scientific and Ethical Committee from Faculty of Pharmaceutical Sciences, University of São Paulo, (protocol number 102/2006, 10 July 2006).

#### 4.1.2. Reagents

Cell culture media and hemoglobin were obtained from Sigma-Aldrich (St. Louis, MO, USA), fetal bovine serum (FBS) from Gibco (Rockville, MD, USA), bovine serum albumin (BSA) (Fraction V) from USB (Cleveland, OH, USA). J774A.1 cells were obtained from ATCC (catalog number TIB-67) and human coronary endothelial cells from Lonza (Basel, Switzerland). CD163 was obtained from R&D Systems, Inc. (Minneapolis, MN, USA). Common Laboratory Chemicals were purchased from Fisher Scientific or Sigma. Peptide was synthesized, per our amino acid sequence plan, by Chinese Peptides Company, (Hangzhou, China) and fully characterized by nuclear magnetic resonance (NMR), carbon, hydrogen and nitrogen (C,H,N) composition analyses and by MALDI/FAB mass spectrometry. ^111^InCl_3_ (Nordion Inc., Ottawa, ON, Canada) was supplied by Nuclear and Energy Research Institute (IPEN)/CNEN, (São Paulo, Brazil). HPLC analysis were performed in a Shimadzu VP series equipment (Shimadzu, Japan) connected to radiation detector Flow Scintillation Analyzer, Radiomatic 610RT (PerkinElmer, Boston, MA, USA).

#### 4.1.3. Atheroma Plaques and Normal Carotid Specimens

We acquired random atherosclerotic plaques (*n* = 23) and normal carotid (*n* = 3) specimens. The inclusion criteria to organs donors (control), it was no more 55 years old and patients over 18 years old. Exclusion criteria included infectious diseases (including AIDS), cancer, surgical intervention in the same area, transplanted or immunosuppressed patients and familiar’s dyslipidemia. Random atherosclerotic plaque specimens were obtained from three patients (male, with ages ranging from 61 to 72 years), diagnosis details are included in Table 1, who underwent carotid endarterectomy procedures. One day before surgery, written informed consent was obtained from the patient or legal next of kin, and the patient was then enrolled in the study. Since endarterectomy does not preserve the tissue integrity, carotid specimens exposed to phage display may compose the whole arterial thickness, the intima or the inner media. A normal carotid specimen was obtained from three healthy organs donors (male, with ages ranging from 16 to 36 years).

#### 4.1.4. Phage Display Selection

A peptide phage display library with seven random amino acids flanked by cysteine residues (labeled as CX7C) with an estimated diversity of 10^8^ peptides was used in the study [8]. A representative fragment of the atherosclerotic plaque (~1 cm^2^) was excised from the specimen, washed 3 times with DMEM free of antibiotics, blocked with DMEM 3% BSA for 15 min at 4 °C (to minimize non-specific phage binding), and then incubated with 10^9^ TU (transducing units) of the CX7C phage library in 100 μL of DMEM 3% BSA. Phage display selection was done directly on the luminal face of the atherosclerotic plaque for 2 h at 4 °C. The atherosclerotic plaque was then washed three times with DMEM and bound phage recovered by infection with log phase *E. coli* K91kan, amplified overnight in LB kanamycin (100 μg/mL) and tetracyclin (20 μg/mL) (LBKan/Tet) to be used in the next round of selection [8]. The total number of phage bound to the specimen in each round of selection was determined by colony counting from small samples plated in triplicate on LBKan/Tet agar (before overnight culture).

Three successive rounds of biopanning were performed after which randomly picked phage clones were selected for sequencing to determine the DNA insert coding the peptide display by the bacteriophage. DNA sequencing was performed using the oligonucleotides 5′-CATGCCCGGGTACCTTTCTATTCTC-3′ (sense) and 5′-CCCTCATAGTTAGCGTAACGATCT-3′ (anti-sense) (Sigma-Genosys Laboratories, St. Louis, MO, USA) to amplify the region containing the DNA insert in the pIII gene, the Cy5 AutoCycle Sequencing Kit (Pharmacia Biotech, Pittsburgh, PA, USA) and an ABI Prism TM 3100 genetic Analyzer. The search for sequence similarity to known human proteins was done using the pBlast software and the Genbank database (http://www.ncbi.nlm.nih.gov).

#### 4.1.5. Phage Amplification

Selected phages were amplified by infection with log phase *E. coli* K91kan followed by overnight culture in 500 mL of LBKan/Tet at 250 rpm. Next day, cell cultures were clarified by centrifugation (8.000× *g* for 15 min), and phage present on the supernatant precipitated with polyethylene glycol (PEG) molecular weight (*M*w) 8000, 2.5% (*w*/*v*) and NaCl (0.38 M) (final concentrations). Phages were resuspended in phosphate buffered saline solution (PBS) at approximately 10^12^ TU/mL and sequenced as described above.

#### 4.1.6. Phage Overlay Assay

Phage binding to carotid atherosclerotic plaque, normal carotid, as well as spleen and thyroid as control specimens, was performed by the phage overlay method [8,24] with modifications. Paraffin-embedded tissue sections (4 μm) were deparaffinized and rehydrated with xylene and graded alcohols, blocked for endogenous peroxidases, and antigen retrieved in a microwave oven by treatment with citrate buffer (pH 6.0) or Tris/EDTA (1 mM, pH 9.0). The slides were then blocked for nonspecific binding with protein block (Dako, Glostrup, Denmark) and 2 × 10^9^ TU of specific phage or Fd-tet phage (negative control) were incubated for two hours at RT with the tissue sections. Phages bound to the tissue were revealed with an anti-bacteriophage antibody (Sigma), peroxidase conjugated secondary antibody and DAB. Slides were counterstained with hematoxylin–eosin (HE). For the spleen and thyroid samples that represent positive and negative CD163 controls respectively, tissue sections were deparaffinized, hydrated, and antigen retrieved as described above with modifications to immunofluorescence and fluorescence microscopy. The spleen and thyroid tissue sections were incubated with anti-CD163 (Novocastra Laboratories, New Castle Upon Tyne, UK) or 2 × 10^9^ TU of specific phage (or Fd-tet phage, negative control) for two hours at RT. Phages bound to tissue were revealed with the anti-bacteriophage antibody followed by anti-rabbit immunoglobulin conjugated to FITC. CD163 expression was detected with an anti-mouse immunoglobulin conjugated to Cy3. Tissue sections were then photographed with an AxioVision (Carl Zeiss Inc., Jena, Germany) microscope.

#### 4.1.7. Quantification of Phage Positivity

The quantification of binding phage to tissues was carried out in twelve samples (*n* = 3, each group), that were divided in four different groups: Atheroma with CTHRSSVVC-phage; Atheroma with Fd-phage; Normal carotid with CTHRSSVVC-phage and Normal carotid with Fd-phage. Both positive and negative intimal areas for the phage in human and animal model were quantified by using automatic detection of colors at a computerized image analysis system Axio Vision (Carl Zeiss Inc., Jena, Germany), coupled to a light microscope with a 10× magnification; the whole sections stained with immunoperoxidase reactions were analyzed. Then the percent area of positivity was calculated in each case: positive areas × 100/(positive + negative areas).

#### 4.1.8. Phage Binding Assay

Binding of selected phage to immobilized receptors was performed as described [14]. Briefly, Hb, Hp and the Hb-Hp complex or recombinant CD163 were immobilized on microtiter plate wells (1 μg in 50 μL) overnight at 4 °C, washed with PBS, blocked with PBS 3% BSA, and incubated with 10^9^ TU of specific phage or Fd-tet phage (negative control). After 2 h, the wells were washed 10 times with PBS and bound phage were recovered by bacterial infection and quantified by colony count on LBKan/Tet plates.

#### 4.1.9. Phage Internalization

The phage internalization assay was performed as described [15,24]. J774A.1 cells or HCAECs (10^4^) were seeded onto micro-chamber glass slides and cultured overnight in DMEM 10% FBS. Next day, the wells were washed with DMEM, blocked with DMEM 30% FBS for one hour as 37 °C, and incubated with 2 × 10^9^ TU of specific phage or Fd-tet negative control phage in 200 μL of DMEM 2% FBS overnight at 37 °C. Cells were then washed three times with PBS, three times with 50 mM Glycine pH 2.8 (adjust with HCl), 150 mM NaCl, buffered solution (two minutes each wash), and three times with PBS. Fixed with 4% paraformaldehyde in PBS and internalized phage detected with a specific anti-bacteriophage antibody (Sigma, St. Louis, MO, USA) in Triton X-100 permeabilized cells.

#### 4.1.10. ^111^In-DOTA-CTHRSSVVC Peptide Preparation and Atheroma/Normal Carotid Binding Studies

Fifty microliters of a solution of ^111^In-DOTA-CTHRSSVVC peptide (0.5 mM) was added to a vial followed by addition of 100 µL of sodium acetate (0.4 M, pH 5) and 2 to 4 µL of ^111^InCl_3_ (74–129.5 MBq), the solution was heated at 70 °C, by 20 min. The product was analyzed by HPLC using a Bondaclone-RP-C18-4.6 × 300 mm × 10 µm, mobile phase (A) H_2_O:TFA 0.1%, phase (B) acetonitrile:TFA 0.1% (100% A to 100% B in 20 min). Ten microliters of labeled solution were diluted in 1 mL saline. Tissues (atheroma and normal carotid, *n* = 2 each) were cut in small fragments, added to four wells plate (as duplicate), and immersed in saline (0.9%). Twenty microliters of peptide were added into wells and stand at the temperature of 4 °C, by 30 min. Afterwards, tissues were removed and washed in three consecutive vials containing saline. Activity in well solution, in the three vials and in the tissue was measured in a gamma counter, and percentage of binding was determined dividing counts in tissues by sum of the total counts.

A blocking experiment was conducted adding 20 µL of 0.5 mM DOTA-CTHRSSVVC peptide, stand at 4 °C for 30 min before the addition of the labeled peptide, then following previously described procedure.

### 4.2. CTHRSSVVC-Phage Targeting Atherosclerotic Lesions in Vivo

#### 4.2.1. Animal Model (LDLr^−/−^)

Homozygous LDL receptor-deficient male mice (C57BL/6J background) were purchased from Jackson Laboratories (Bar Harbor, ME, USA) and maintained in the Faculty of Pharmaceutical Sciences (The University of São Paulo, São Paulo, Brazil) in accordance with institutional guidelines. Mice between 3 and 5 months of age were maintained in individual plastic cages at 22 °C on a 12-h light–dark cycle. A total of 21 male LDLr^−/−^ mice, were divided into two groups: (1) High-Fat diet group (*n* = 13) and (2) Regular-diet group (*n* = 8) for 35 days. The High-Fat diet consisted in a semisynthetic chow based on a Western-type diet containing 20% fat, 0.5% (*w*/*w*) cholesterol (Sigma-Aldrich, St. Louis, MO, USA), 0.5% (*w*/*w*) cholic acid (Sigma-Aldrich), 16.5% casein, vitamins and minerals, according to the recommendations of AIN-93 [37]. The Regular-diet consisted in the standard chow according to the National Research Council recommendations.

The animals were treated in strict accordance with the National Institutes of Health Guide for the Care and Use of Laboratory Animals (NIH Publication N 85–23, revised in 1996), and received water and chow ad libitum throughout the experiment.

#### 4.2.2. CTHRSSVVC-Phage Homing in LDLr^−/−^ Mice

For homing of selected CTHRSSVVC-phage in vivo, after 35 days receiving the chows, animals were anaesthetized and received the systemic injection of 1 × 10^9^ TU targeted phage, insertless control phage or vehicle, in 50 μL of saline 0.9%, into the tail vein. After 24 h of circulation, the animals were anaesthetized with a mixture of 2:1 of ketamine (1.0 g/10.0 mL; Vetaset, Fort. Dodge Saúde Animal Ltda, Campinas, São Paulo, Brazil) and xylazine (2.0 g/100 mL; Bayer do Brasil, Brazil) before euthanasia. The heart was perfused with phosphate buffered saline (PBS) and 10% PBS-buffered formaldehyde. The heart and aortas were excised, fixed in 10% formaldehyde for at least two days and forwarded to be paraffin-included. No heart was excised to quantification of targeted phage particles recovered in the aortic root.

#### 4.2.3. Immunohistochemistry

Paraffin-embedded tissue sections (4 μm) were deparaffinized and rehydrated with xylene and graded alcohols, blocked for endogenous peroxidases, and antigen retrieved in a microwave oven by treatment with citrate buffer (pH 6.0) or Tris/EDTA (1 mM, pH 9.0). The slides were then blocked for nonspecific binding with protein block (Dako, Glostrup, Denmark), and a polyclonal rabbit anti-bacteriophage primary antibody (Sigma) was added (1:500 dilution), followed by 1-h incubation with a peroxidase conjugated anti-rabbit secondary antibody. Slides were developed with a specific substrate (chromogen DAB) and counterstained with hematoxylin–eosin (HE). As a reaction control, primary antibody was omitted. Tissue sections were then photographed with an AxioVision microscope.

### 4.3. Statistical Analysis

The quantification of binding phages to human samples was performed with the use of Graph Prisma 5.0, and statistical significance was determined by one-way analysis of variance followed by the Kruskal–Wallis test, and it was accepted at a value of *p* < 0.05. The statistical significance of experimental observations was determined by Fisher´s exact test at a value of *p* < 0.01 as statistically significant. The phage bound quantification to High-Fat diet group with CTHRSSVVC-phage are reported as means ± SEM ± SD, the percent of positivity area was calculated in each case: positive areas × 100/(positive + negative areas).

## 5. Conclusions

In summary, the results of our investigation suggest that the CTHRSSVVC synthetic peptide is a CD163-mimic peptide, which maps to a putative homodimerization domain, and binds to human and mouse atherosclerotic lesions [18]. Further studies are ongoing to expand the scope of this peptide using nanotechnology and radiolabeling tools to develop new diagnostic and therapeutic approaches in treating cardiovascular diseases. Gaining insights on the role of fifth SRCR domain of CD163 to atherosclerosis and how other diseases, as well as the macrophage polarity, influences the specific uptake of this peptide would be of paramount importance.

## Figures and Tables

**Table 1:** Phage overlay in atherosclerotic and normal carotid tissue.

Data Descriptions	Histopathology	Controls		Selected Phages			
Peptides		NPA	Fd	CVSSTLLRC	CVQLNSLPC	CQAYKLGSC	CTHRSSVVC
Similarity				PlGF	Galectin 4	Collagen α-1 (IX) chain	CD163
Tissues/Diagnosis							
Patient 1 Hypertension, dyslipidemia and aneurysm	Inflammation, without thrombus, calcium, no hemorrhage.	−	−	+ FC	NP	NP	++ LC
Patient 2 Diabetes and revascularization	Inflammation, thrombus, calcium, hemorrhage.	−	−	+ FC	+ FC	+ FC	++ LC, T
Patient 3 Revascularization, hypertension, dyslipidemia, stroke and abdominal aortic aneurysm	Inflammation, thrombus, calcium, no hemorrhage.	−	−	+ FC	+ FC	+ FC	++ LC, T
Non Atherosclerotic Carotid 3	Normal	−	−	−	−	NP	−

NPA = no phage addition, Fd = wild phage, (−) = negative, (+) = low positivity, (++) = strong positivity, FC = fibrous cap, NP = not performed, LC = lipid core, T = thrombus.

**Table 2:** Human homolog for select atheroma binding peptides.

Peptide	Motif	Blast Score *	Protein	GenBank
CVQLNSLPC	QLNSLP	21.8	Galectin 4	AAH34750.1
CQAYKLGSC	AYKLGS	20.3	Collagen α-1 (IX) chain	NP_001094312.1
CTHRSSVVC	C+B/A+HSR-VVC	38.8	CD163	CAB45233.1
CVSSTLLRC	CVS LLRC	18.9	Placental growth factor	AAB30462.2
CQALSNALC	QALSNAL	20.6	Angiomotion	AAI30295.1
CDLLYNGVC	DLLYN+C	23.1	Ephrin type-B receptor 3	NP004434.2
	DLL+NVC	21.8	Ephrin type-B receptor 6	ABV55388.1
CGTQSGASC	T+SGASC	21.0	Tissue plasminogen activator	CAX11668.1
CRREGVERC	R+GVERC	24.0	Emilin-1	AAH09947.2
CDGRFVRVC	RFVRV	19.7	Apolipoprotein L4	CAQ08533.1
CFVAGRVRC	FVA RVR	21.4	Inhibin β C	NP_005529.1
CLEDSSWAC	CLED WA	22.7	C4b-binding protein β	NP_001017364.1
CTSVVSSRC	TSVVSSR	23.1	Integrin α-11	AAD51919

Peptides sequences isolated by ex vivo phage display library were analyzed using BLAST* (NCBI) to search for similarity to known human proteins.

**Table 3:** Quantification of phage positivity for human atheroma samples.

Samples	* Atheroma CTHRSSVVC-Phage	Atheroma Insertless-Phage	Normal Carotid CTHRSSVVC-Phage	Normal Carotid Insertless-Phage
1	10.14	0.01	0.01	0.00
2	10.88	0.00	0.09	0.00
3	16.02	0.00	0.01	0.00
±SEM	12.35	0.00	0.04	0.00
±SD	3.20	0.01	0.05	0.00

Statistical difference was taken into consideration when * *p* < 0.05, vs. Normal Carotid Insertless-phage.

**Table 4:** Phage homing to atherosclerotic lesions at aortic root of LDLr^−/−^ mice.

Phages/Control	LDLr^−/−^ High-Fat Diet	LDLr^−/−^ Regular-Diet
CTHRSSVVC-phage *	100% (*n* = 6)	0% (*n* = 4)
Insertless phage (Fd)	0% (*n* = 6)	0% (*n* = 3)
Vehicle Total	0% (*n* = 1) (*n* = 13)	0% (*n* = 1) (*n* = 8)

LDLr^−/−^ were fed High-Fat or Regular diet. * *p* < 0.01 compared to Regular-diet by Fisher’s test CTHRSSVVC-phage * to LDLr^−/−^ mice High-Fat diet homing 100% positivity.

## Supplementary Materials

Supplementary materials can be found at www.mdpi.com/1422-0067/17/9/1383/s1.
