# Supplementary material for: CTHRSSVVC Peptide as a Possible Early Molecular Imaging Target for Atherosclerosis
Source: Int J Mol Sci. 2016 Aug 24;17(9):1383. doi: 10.3390/ijms17091383 (PMC5037663; doi:10.3390/ijms17091383)
Supplement: Supplementary file 1 [file ijms-17-01383-s001.pdf]

Supplementary Materials: CTHRSSVVC Peptide as a Possible Early Molecular Imaging Target for Atherosclerosis

Rosemeire A. Silva, Ricardo J. Giordano, Paulo S. Gutierrez, Viviane Z. Rocha, Martina Rudnicki, Patrick Kee, Dulcineia S. P. Abdalla, Pedro Puech-Leão, Bruno Caramelli, Wadih Arap, Renata Pasqualini, Jose C. Meneghetti, Fabio L. N. Marques, Menka Khoobchandani, Kattesh V. Katti, Ademar B. Lugão and Jorge Kalil

1. Materials and Methods

^{111}In-DOTA-CTHRSSVVC Synthetic Peptide Homing in LDLr$^{-/-}$ Mice

The animals received water and chow ad libitum throughout the experiments and were treated in accordance with the National Institutes of Health Guide for the Care and Use of Laboratory Animals (NIH Publication N 85–23, revised in 1996). The animal procedures to this pilot study were reviewed and approved by the Scientific and Ethical Committee from Faculty of Pharmaceutical Sciences, University of São Paulo (protocol number CEUA/FCF/266/2010, 04/10/2010).

Homozygous LDL receptor-deficient male mice (C57BL/6J background) were purchased from Jackson Laboratories (Bar Harbor, ME, USA) and maintained in the Faculty of Pharmaceutical Sciences (The University of São Paulo, São Paulo, Brazil) in agreement with institutional guidelines. Mice aged 3–5 months were maintained in individual plastic cages at 22 °C on a 12-h light–dark cycle. A total of four male LDLr$^{-/-}$ mice were divided into two groups: (1) High-Fat diet group ($n = 2$); and (2) Regular-diet group ($n = 2$) for 12 months. The High-Fat diet consisted of a semisynthetic chow based on a Western-type diet containing 20% fat, 0.5% (*w*/*w*) cholesterol (Sigma-Aldrich, St. Louis, MO, USA), 0.5% (*w*/*w*) cholic acid (Sigma-Aldrich, St. Louis, MO, USA), 16.5% casein, vitamins and minerals, according to the recommendations of AIN-93 [37]. The Regular-diet consisted of the standard chow according to the National Research Council recommendations.

After 12 months receiving the chows, animals were anaesthetized with a mixture of 2:1 of ketamine (1.0 g/10.0 mL; Vetaset, Fort. Dodge Saúde Animal Ltda, Campinas, São Paulo, Brazil) and xylazine (2.0 g/100 mL; Bayer do Brasil, Brazil) and received systemic injection of ^{111}In-DOTA-CTHRSSVVC synthetic peptide (26 MBq/nmol) in 50 µL of saline 0.9 %, into the penile vein. After seven hours, animals were anaesthetized, euthanized and organs excised. Arteries were stored in a freezer (−20 °C) for 48 h. The arteries images were taken in a micro-SPECT camera (Triumph Trimodality-Gamma Medica-Ideas, Northridge, CA, USA); after additional storage in freezer for seven days, arteries were imagined by autoradiography (StormTM-Molecular Dynamics, Sunnyvale, CA, USA).

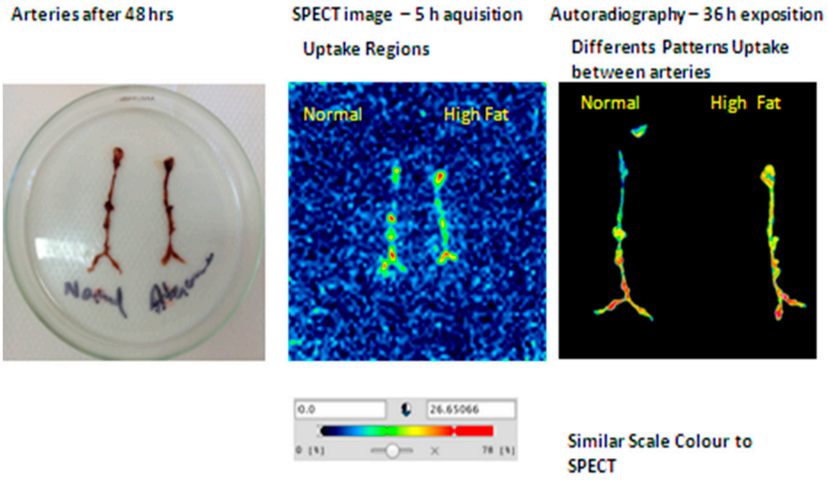

Figure S1. Single-photon emission computed tomography (SPECT) and autoradiography pre-clinical images. (Magnification = 1×).

2. Conclusions

We have performed in vivo LDLr$^{-/-}$ mice pilot image using ^{111}In-DOTA-CTHRSSVVC synthetic peptide. However, the results are still preliminary as we did not have sufficient samples to allow for statistical analysis. SPECT images of excised organs and autoradiography of Normal and High-Fat diet LDLr$^{-/-}$ mice arteries were performed. The autoradiography clearly demonstrated clinically discernable uptake patterns. It is also important to note that ^{111}In is not the best radionuclide of choice for imaging purposes. Additional imaging experiments using PET radionuclides are underway and will be reported in future publications.
